# Double Ulcer of Stomach and Duodenum

**Published:** 1914-08

**Authors:** 


**Double Ulcer of Stomach and Duodenum,** Pauchet of Amiens, Le Progress Med., May 8, 1914. Woman, aged 31, symptoms of duodenal ulcer from 14 to 20, of gastric ulcer from 20 to

28, stenosis with stasis and copious vomiting for 3 years. Radiologic diagnosis of hour-glass stomach and duodenal stenosis, confirmed by operation, consisting of anastomosis of the two pouches of the stomach and the lower one with the duodemum. Good results for 18 months, gain of weight of 33 pounds (literally, patient enfats of 15 kilos). On account of corrosion of abdominal wall (by adhesion?) and peptic ulcers with retraction at each anastomosis, a second operation was performed after two years. Resection of stomach and duodenum, except part of the upper pouch, peritoneal lavage with ether, the latter causing grave toxic symptoms and death after three or four hours.

